# The role of BCAA metabolism in metabolic health and disease

**DOI:** 10.1038/s12276-024-01263-6

**Published:** 2024-07-02

**Authors:** Byeong Hun Choi, Seunghoon Hyun, Seung-Hoi Koo

**Affiliations:** Division of Life Sciences, Korea University, Seoul, Korea

**Keywords:** Metabolic disorders, Ageing

## Abstract

It has long been postulated that dietary restriction is beneficial for ensuring longevity and extending the health span of mammals, including humans. In particular, a reduction in protein consumption has been shown to be specifically linked to the beneficial effect of dietary restriction on metabolic disorders, presumably by reducing the activity of the mechanistic target of rapamycin complex (mTORC) 1 and the reciprocal activation of AMP-activated protein kinase (AMPK) and sirtuin pathways. Although it is widely used as a dietary supplement to delay the aging process in humans, recent evidence suggests that branched-chain amino acids (BCAAs) might be a major cause of the deteriorating effect of a protein diet on aging and related disorders. In this review, we delineate the regulation of metabolic pathways for BCAAs at the tissue-specific level and summarize recent findings regarding the role of BCAAs in the control of metabolic health and disease in mammals.

## Dietary Restriction Benefits: BCAA’s Influence on Aging and Health

This review article illustrates the function of branched-chain amino acids (BCAAs - essential nutrients we get from food) and how they’re processed in our bodies, in relation to health and illness. BCAAs are connected to aging processes and metabolic health - the body’s way of converting food into energy. Recent studies found that reducing BCAA intake can improve the health and lifespan of rodents. Similar studies were also conducted by using different animal models, like yeast, flies, rodents, and primates. It also emphasized the potential influence of BCAAs on human disease and aging metabolic processes. The review article concluded that BCAAs and their processing are vital for metabolic health and lifespan, and more research is needed to understand their effect on human health. Further studies on BCAAs could be important for creating diet plans and treatments for metabolic issues and aging-related diseases.

This summary was initially drafted using artificial intelligence, then revised and fact-checked by the author.

## Introduction

Dietary restriction (DR) has long been associated with longevity and improved overall metabolism in various animal models, including yeast, fly, rodent, and primate models such as rhesus monkeys^1–3^. Reducing dietary intake not only reduces the accumulation of fats in adipose tissue but also affects cellular signaling pathways that increase lifespan and inhibit the aging process. Key regulators that are directly affected by DR are known to be nutrient sensors, such as the mechanistic target of rapamycin complex (mTORC) 1, Sirt1 and AMP-activated protein kinase (AMPK), which are considered to be regulators of aging and age-associated metabolic pathways^4–6^.

Specifically, the beneficial effect of DR could be linked to a reduction in protein intake. Indeed, recent studies revealed that the ratio of dietary protein to carbohydrates, but not DR itself, could be a critical determinant of the effect of a controlled diet on longevity and metabolic health, at least in rodent models^7^. Thus, efforts have been made to identify specific amino acids of proteins that are linked to this pathway in recent years. Among the individual amino acids, branched-chain amino acids (BCAAs) are mainly associated with aging-related pathways and metabolic health^8–11^. BCAAs constitute ~25% of the amino acids in individual proteins in humans and should be obtained from foods. Therefore, BCAAs are considered critical essential amino acids^12^. Interestingly, despite the importance of BCAAs as a key protein component, restriction of BCAA intake increases longevity and health span in rodents^10^, suggesting that BCAAs or their metabolic pathways could be critical in stimulating the aging process and decreasing metabolic health in mammals.

In this review, we explore the potential role of BCAAs and their metabolism in the regulation of human disease and age-associated metabolic pathways by mainly focusing on metabolic tissues.

## The branched-chain amino acids

### Metabolic pathways of BCAAs

BCAAs consist of leucine, isoleucine, and valine, which are three major essential amino acids. After the consumption of a protein diet, digested BCAAs in the intestinal lumen are taken up by Na^+^-dependent transporters of the apical membrane of intestinal epithelial cells. They are then released to the bloodstream by facilitated transport and are specifically transported across cell membranes of tissues mainly by the L-type Na^+^-dependent cotransporter LAT1 and its heterodimeric partner 4F2hc^13–16^.

**Fig. 1:**
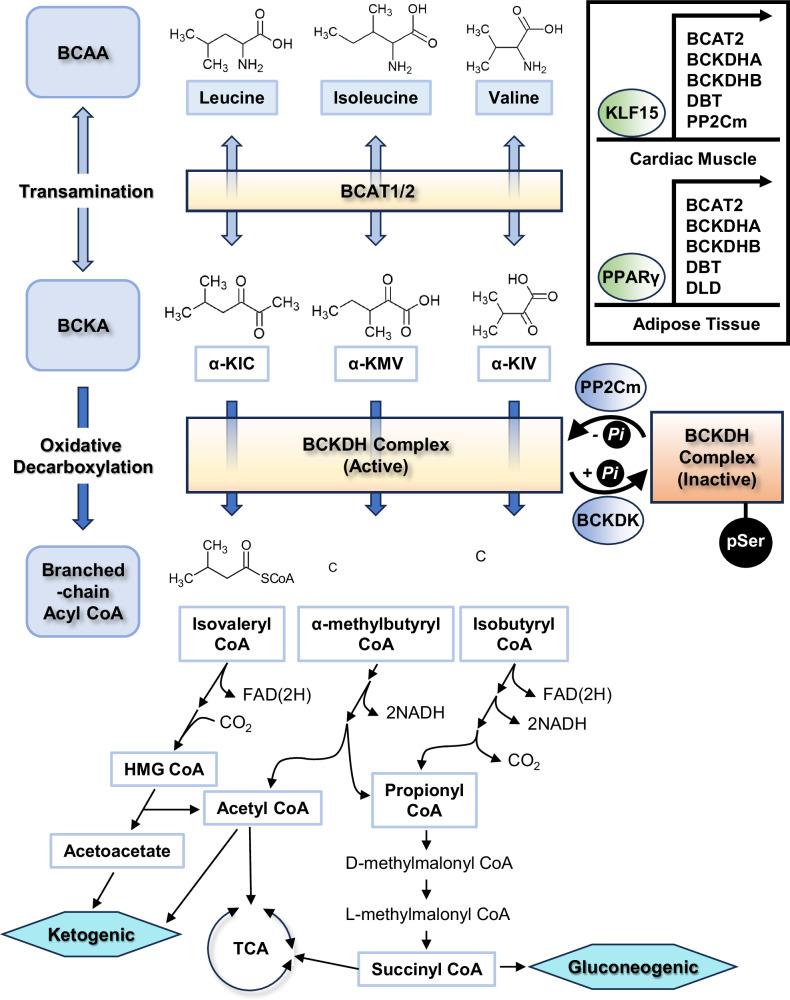
Schematic representation of BCAA catabolism. The first step of BCAA catabolism is the transamination of BCAAs by BCAT1/2 to form BCKA. BCKAs can be transformed to BCAAs by the same enzyme. Subsequently, BCKAs are converted into branched-chain acyl-CoA by the BCKDH complex. Branched-chain acyl-CoA is then further oxidized to acetyl-CoA and propionyl CoA, which can then enter the TCA cycle. The activity of the BCKDH complex is regulated via the phosphorylation of the E1 enzyme by BCKDK and PP2Cm. (Box) Transcriptional regulation of BCAA catabolic enzyme-encoding genes by KLF15 (cardiac muscle) and PPARγ (adipose tissues). BCAA branched-chain amino acid, BCKA branched-chain keto acid, BCAT branched amino acid aminotransferase, BCKDH branched-chain keto acid dehydrogenase, BDKDK BCKDH kinase, PP2Cm protein phosphatase 2Cm, KLF15 Krȕppel-like factor 15, PPARγ peroxisome proliferator-activated receptor γ.

The first two steps of BCAA degradation are carried out by branched amino acid aminotransferase (BCAT) and branched-chain keto acid dehydrogenase (BCKDH). BCAT consists of two different isozymes, BCAT1 and BCAT2^17,18^ (Fig. 1). BCAT1 is a cytosolic enzyme found only in limited tissues, such as embryonic tissues, the brain, and the ovary. However, the mitochondrial enzyme BCAT2 is ubiquitously expressed in various tissues, including skeletal muscle and adipose tissue. BCAT acts upon all three BCAAs and converts them into branched-chain keto acids (BCKAs) and glutamate. In the next step, BCKAs are converted into distinct forms of branched-chain acyl-CoA by the BCKDH complex^19^. The BCKDH complex is an α-keto acid dehydrogenase complex that includes a pyruvate dehydrogenase complex and an α-ketoglutarate dehydrogenase complex^20,21^. As with other α-keto acid dehydrogenase complexes, the BCKDH complex consists of three subunits: E1 α-keto acid decarboxylase, E2 transacylase, and E3 dihydrolipoyl dehydrogenase. Several cofactors are involved in the enzymatic activity of this complex. Thiamine pyrophosphate on the E1 subunit decarboxylates BCAA and forms an intermediate with the remaining acyl group. The acyl group is then moved to the E2 subunit by lipoate and is subsequently transferred to CoA to produce branched-chain acyl-CoA. Finally, the reduced lipoate is reoxidized by the E3 enzyme, which contains flavin adenine dinucleotide (FAD) as a cofactor. The electrons removed from lipoate are moved to the oxidized form of nicotine amide dinucleotide (NAD^+^) via FAD, resulting in the formation of the reduced form of nicotine amide dinucleotide (NADH).

In subsequent steps, branched-chain acyl-CoA is degraded in parallel^12^. Isobutyryl CoA, generated from the catabolic pathway of valine, is eventually converted to propionyl CoA and enters the tricarboxylic acid (TCA) cycle via a form of succinyl CoA. During the degradation of isoleucine, the intermediate 2-methylbutyryl CoA is converted to acetyl-CoA and propionyl CoA, each of which can eventually join the TCA cycle. Finally, isovaleryl CoA, an intermediate of leucine catabolism, is converted to β-hydroxy-β-methylglutaryl CoA (HMG CoA), which is eventually converted to acetyl-CoA. The degradative pathway for these branched-chain acyl-CoA molecules also involves the generation of NADH and FAD(2H). Thus, BCAA catabolism could function as a key pathway for the generation of energy by mitochondrial oxidative phosphorylation. Alternatively, BCAA catabolites could funnel into the generation of fatty acids, cholesterol, ketone bodies or glucose depending on the nutritional status or the specific needs of the cells and organisms.

### Regulation of the BCAA catabolic pathway

Like that of many metabolic pathways, the regulation of BCAA catabolism occurs mainly in its rate-limiting step, the conversion of BCKA into branched-chain acyl-CoA by the BCKDH complex. This complex is located in the inner mitochondrial membrane, and its activity is allosterically inhibited by its major products, branched-chain acyl-CoA and NADH. In addition, the phosphorylation status of this complex dictates its activity. BCKDH kinase (BCKDK) catalyzes the phosphorylation of serine 293 of BCKDHA, which is a component of the BCKDHA/BCKDHB heterodimer that constitutes the E1 subunit. Therefore, this phosphorylation inhibits the activity of the BCKDH complex. Conversely, protein phosphatase 2Cm (PP2Cm) functions as a specific phosphatase that removes a phosphate from serine 293 of BCKDHA, thus activating the BCKDH complex^22,23^.

Recent studies revealed that the chronic regulation of BCAA catabolism is achieved via the transcriptional regulation of gene expression^24^. Krȕppel-like factor 15 (KLF15) belongs to the family of KLF proteins that bind the 5’-CACCC-3’ motif in the promoter of their target genes^25^. It is expressed ubiquitously and is involved in adipose tissue functions, insulin secretion in islets, and the regulation of skeletal and cardiac muscle functions^26–29^. Interestingly, in cardiac muscle, KLF15 has been shown to be involved in the transcriptional activation of various genes involved in BCAA catabolism, including BCAT2, BCKDHA, BCKDHB, DBT (which encodes the E2 subunit of the BCKDH complex), and PP2Cm, suggesting a role for the KLF15-BCAA catabolism axis in the control of cardiac function^30^. The role of KLF15 in the control of BCAT2 expression was also verified in other tissues, including liver and skeletal muscle^31,32^. The role of KLF15 in individual tissues should be more carefully assessed by using tissue-specific and/or ligand-specific KLF15 knockout mice to assess the potential effect of KLF15 depletion on the developmental process.

Peroxisome proliferator-activated receptor (PPAR) γ belongs to a family of PPAR transcription factors, a subfamily of nuclear hormone receptors^33^. Like its family members, it forms a heterodimer with the retinoid X receptor (RXR) on the direct repeat of the 5’-AGGTCA-3’ sequence on the promoter of target genes. First identified as a master regulator of adipogenesis, it is widely considered a major therapeutic target for type 2 diabetes (T2D) in part via its ability to enhance insulin sensitivity. Indeed, thiazolidinediones (TZDs), which are synthetic agonists of PPARγ, have been shown to enhance insulin sensitivity, TZDs were used to treat T2D until recently, after which they were taken off the market due to various complications, including heart failure. In addition to its role in adipogenesis, PPARγ was also shown to regulate BCAA metabolism, at least in adipose tissue. Treatment with TZD increased the expression of genes involved in BCAA catabolism, such as BCAT2, BCKDHA, BCKDHB, DBT and DLD (which encode the E3 complex of the BCKDH complex), in human adipose tissue^34^. Similarly, in Zucker obese rat models, treatment with TZD dramatically improved insulin sensitivity and increased the expression of genes involved in BCAA catabolism, such as BCKDHB, DBT and DLD, in adipose tissue^35^. These findings suggest that BCAA catabolism in adipose tissue could be transcriptionally regulated by PPARγ in mammals. Indeed, the expression of genes involved in BCAA catabolism was dramatically reduced in inguinal adipose tissue and brown adipose tissue (BAT) in PPARγ-depleted mice compared with wild-type controls^36^, corroborating the role of this transcription factor in controlling BCAA catabolism in adipose tissue. Further study is necessary to verify the role of PPARγ in BCAA catabolism in other cell types that express this transcription factor, such as hepatocytes or macrophages. In addition, the potential role of other PPAR family members in regulating BCAA catabolism should also be explored to assess the potential crosstalk between transcription factors. These possibilities could be easily explored by analyzing publicly accessible transcriptomics databases.

## Role of BCAA catabolism in cellular physiology

### Skeletal muscle and liver

The activity of BCAT and BCKDH is highest in skeletal muscle, followed by adipose tissue and the brain; thus, skeletal muscle is a major site for BCAA catabolism^37^. In conjunction with the role of BCAA catabolism in providing energy via mitochondrial oxidative phosphorylation, exercise was shown to strongly increase BCAA catabolism in skeletal muscle in rodents^38,39^. Thus, theoretically, the skeletal muscle could catabolize the majority of BCAAs for its own use and supply intermediates of the BCAA catabolic pathway (e.g., BCKA) to other tissues. Due to the low expression of BCATs and relatively high expression of BCKDH in the liver, BCKAs originating from skeletal muscle can be utilized in the liver. For example, they can be utilized in catabolic pathways to produce ATP via mitochondrial oxidative phosphorylation and the production of lipids such as fatty acids and cholesterol. In addition, α-ketoisocaproic acid (BCKA from leucine) and α-ketomethylvaleric acid (BCKA from isoleucine) can be utilized to generate glucose (via gluconeogenesis) and ketone bodies depending on metabolic needs during BCKA catabolism^11^.

### Adipose tissues

A recent study revealed a critical role for adipose tissue in BCAA catabolism. BCATm knockout mice lacking BCAT2 expression in all tissues displayed increased plasma BCAA levels^40^. Transplanting wild-type adipose tissue into BCATm knockout mice decreases plasma BCAA levels by 30–50%, suggesting that BCAA utilization or catabolism in adipose tissue is also critical for maintaining BCAA homeostasis at the systemic level. Among adipose tissues, BAT plays a specific role in the regulation of body temperature by controlling adaptive thermogenesis. A recent study showed that BAT utilizes BCAA to control adaptive thermogenesis in response to cold exposure^41^. SLC25A44, which transports BCAAs into mitochondria, is essential for BCAA catabolism in BAT. Furthermore, the authors showed that BCAA catabolism in BAT is essential for the metabolic health of the organism by controlling thermogenic capacity, whole-body BCAA homeostasis, and systemic energy expenditure.

### Brain

The brain is protected by its highly selective blood‒brain barrier (BBB) to preserve its optimal biochemical environment. Interestingly, BCAAs can readily cross the BBB mainly via the universal LAT1/4F2hc and, to a lesser extent, via other L-type carriers, such as LAT2-4^42^. In the brain, BCAAs are involved in key cellular functions, such as glutamate metabolism, protein synthesis and the generation of energy. As a primary excitatory neurotransmitter, the presence of glutamate is crucial for the maintenance of brain function. However, its inability to traverse the BBB easily necessitates its synthesis within neurons. Interestingly, approximately one-third of glutamate in the brain contains nitrogen from BCAAs, suggesting that BCAA metabolism in the brain is essential for the maintenance of neuronal glutamate levels^43^. Gamma-aminobutyric acid (GABA) is a major inhibitory neurotransmitter that is derived from the decarboxylation of glutamate, and thus, BCAAs are also essential for the generation of this neurotransmitter. Thus, the preservation of BCAA catabolism in the brain is essential for the proper functioning of the central nervous system through the maintenance of key neurotransmitters.

### Systemic control of BCAA catabolism

Recently, systemic BCAA catabolism in normal mice and insulin-resistant mice was quantified via in vivo isotope tracing^37^. BCAA catabolism linked to the TCA cycle was found in most tissues, including skeletal muscle, BAT, kidney, liver, heart, and pancreas, suggesting that BCAA or its metabolites (e.g., BCKA) are utilized at the systemic level. Interestingly, in healthy rodents, insulin or pharmacological activation of BCKDH increases BCAA catabolism in skeletal muscle. In an insulin-resistant state, however, BCAA catabolism is almost completely blocked in adipose tissue and the liver and is shifted to cardiac and skeletal muscle, resulting in BCAA dysmetabolism even in these tissues. These results suggest that the metabolic state is critical for controlling systemic BCAA catabolism.

## Disease associated with BCAA catabolism

### Maple syrup urine disease

Maple syrup urine disease (MSUD) is an autosomal recessive genetic disorder that originates from mutations in genes for the BCKDH complex, such as BCKDHA/B, DBT, and DLD^44^. As a result of the production of the defunct BCKDH enzyme complex, the plasma levels of BCAA and its immediate catabolite BCKA are elevated. The first sign of this disorder is a distinctive maple syrup-like odor in the patient’s urine due to elevated BCKA levels in the urine. If untreated, symptoms begin to emerge within the first few days of life, including increased neurological dysfunction, such as lethargy, and depending on the severity of the disease, even seizures and coma. Progressive brain damage ensues that will eventually lead to death within months.

BCAA catabolism is critical for the maintenance of glutamate, a crucial neurotransmitter for brain development and cognitive functions in the brain (Table 1). Dysregulation of BCAA catabolism can result in the depletion of glutamate and its derivatives, leading to various neurological dysfunctions. In addition, the accumulation of leucine could cause swelling of the brain due to impaired water homeostasis. This impairment leads to the accumulation of oxidative stress and a reduction in tyrosine levels in the brain, ultimately resulting in impaired protein signaling. Finally, the accumulation of a-ketoisocaproic acid, a BCKA derived from leucine, could contribute to the encephalopathic syndrome.

**Table 1:** Disease associated with BCAA dysmetabolism.

Disease	Mechanism	Therapy and treatment options
MSUD	• Accumulation of leucine could cause swelling of the brain due to osmotic pressure. • Reduction in tyrosine levels in the brain leading to impaired protein signaling. • Accumulation of a-ketoisocaproic acid could contribute to encephalopathic syndrome. • Depletion of glutamate leads to neurotransmitter deficiency.	• Limited BCAA containing diet • Liver transplantation
Heart diseases	• Elevated levels of the mTORC1 activator leucine lead to cardiac hypertrophy. • Increased BCKA levels lead to mitochondrial respiratory issues, inducing ROS accumulation, and ultimately resulting in heart failure.	• NaPB treatment
Obesity, insulin resistance and diabetes	• BCAA level is negatively correlated with insulin sensitivity. • Increased levels of 3-HIB, a catabolite of valine, promotes fatty acid accumulation in muscle and further aggravates the insulin resistance state. • BCKDK worsens lipid metabolism and insulin resistance via regulation of ATP citrate lyase activity.	• NaPB treatment • BCAA-restricted diet

Patients with this disorder should maintain a specialized diet with limited BCAA content supplemented with a BCAA-free amino acid mixture^45^ (Table 1). Thus, patients can avoid the development of unwanted neurological problems, as stated above. Reintroduction of BCKDH activity by transplantation of the liver from normal individuals was also beneficial, at least in pediatric patients, suggesting that systemic restoration of BCAA catabolism could be sufficient to treat MSUD patients at early ages^46^ (Table 1).

### Heart diseases

Impaired BCAA catabolism is also associated with heart disease. Abnormal BCAA catabolism is associated with myocardial infarction and an increased risk of cardiovascular diseases in humans^47,48^. Deficiencies in BCAA catabolism promote heart failure in mouse models, suggesting the causal role of this metabolic pathway in the physiological function of the normal heart^30^. The expression of rate-limiting enzymes involved in BCAA catabolism, as well as that of PP2Cm, was reduced in cardiomyopathy hearts in mouse models. Furthermore, pharmacological activation of BCAA catabolism significantly alleviated postmyocardial infarction-associated dysfunction in the heart of mice^49^. In this study, the authors utilized BT2 (3,6-dichlorobenzo(b)thiopene-2-carboxylic acid), a BCKDK inhibitor, to promote BCKDH-mediated BCAA catabolism in myocardial infarction (MI)-operated mice, suggesting that restoring BCAA catabolism could ameliorate cardiomyopathy in humans. BCAAs, especially leucine, could be signaling molecules that are directly responsible for the pathophysiology of heart disease.

Leucine is a potent activator of mTORC1 that is responsible for cellular growth and anabolic pathways^50^. In addition, inhibition of mTORC1 activity by rapamycin relieves cardiac hypertrophy^51^, suggesting a potential causal role of disturbed BCAA catabolism and the development of heart disorders via activation of the mTORC1 pathway. Indeed, the notion that cardiac BCAA accumulation is responsible for cardiac dysfunction through activation of the mTORC1 pathway was verified in mouse models in a recent study^49^ (Table 1). Alternatively, elevations in BCKA levels could be responsible for the progression of disease in the heart. Elevated BCKA levels in the heart were observed in mouse models of PP2Cm deficiency, which displayed early onset of heart failure. The authors found that BCKAs could promote the induction of reactive oxygen species (ROS) in part due to dysfunctional mitochondrial respiration, suggesting that the BCKA-ROS axis could contribute to the progression of heart disease^30^ (Table 1).

Regardless of the underlying mechanism, restoring BCAA catabolism in the heart could be beneficial for the treatment of heart disease in humans. Although BT2 is not suitable for human use, sodium phenylbutyrate (NaPB), a prescribed drug for patients with urea cycle disorder, was shown to effectively lower plasma BCAA levels in humans by inhibiting BCKDK^52^ (Table 1). Whether NaPB treatment is effective in relieving symptoms of heart disease in humans is of great interest.

### Obesity, insulin resistance, and diabetes

Plasma BCAA levels were first shown to be positively correlated with obesity and insulin resistance in an earlier study^53^. Recent studies have indicated that elevated plasma BCAA levels are evident in obese, insulin-resistant individuals both in humans and rodent models^54–57^ and are linked to related metabolic disorders such as diabetes and cardiovascular diseases^58–62^. Indeed, in weight loss trials in humans, decreased plasma BCAA levels are positively correlated with increased insulin sensitivity, suggesting a potential role for the BCAA metabolic pathway in obesity and insulin resistance^63,64^. As with other metabolic pathways, BCAA catabolism itself is affected by insulin resistance in metabolic tissues, which in turn exacerbates systemic BCAA dysmetabolism in the disease state.

As stated previously, BCAA catabolism was impaired in the adipose tissue of insulin-resistant mice due to the reduced expression of BCAA catabolic enzymes^37,57,65^. BCAA catabolism further shifted into cardiac and skeletal muscle, which increased the metabolic burden of these tissues. Conversely, disrupting BCAA catabolism in BAT promotes insulin resistance and obesity in mice, suggesting that BCAA catabolism in adipose tissue is critical for maintaining overall systemic metabolic homeostasis. Increased BCAA flux in skeletal muscle during insulin resistance exacerbates metabolic disorders in these tissues. 3-Hydroxyisobutyrate (3-HIB) is a catabolite of valine that is associated with lipotoxicity. 3-HIB was shown to function as a regulator of trans-endothelial fatty acid transport, which promotes fatty acid uptake into skeletal muscle^66^ (Table 1). Thus, increased BCAA catabolism in skeletal muscle in turn promotes fatty acid accumulation in the tissue, further aggravating the insulin-resistant state.

Although the liver is responsible for significantly less BCAA catabolism than skeletal muscle or adipose tissue, dysregulation of hepatic BCAA catabolism is also connected with impaired lipid metabolism and insulin resistance. A fructose diet induces the expression of the transcription factor ChREBP-β, which induces the expression of BCKDK, thus reducing BCAA catabolism in the liver. Reducing BCKDK activity not only restored BCAA catabolism but also improved glucose tolerance in rodents in part by directly regulating ATP citrate lyase activity, which indicates that impaired hepatic BCAA catabolism is linked to lipid metabolism and insulin resistance^8^ (Table 1). Thus, restoring BCAA catabolism during insulin resistance could be a potential therapeutic means for combating this metabolic disorder. Indeed, a recent study showed that NaPB treatment effectively improved peripheral insulin sensitivity and glucose homeostasis in T2D patients^67^ (Table 1). Determining the specific mechanism by which NaPB affects local and/or systemic BCAA catabolism would be helpful for the further development of this therapeutic strategy in the future.

## Potential role of BCAA catabolism in influencing healthspan

### Benefits of BCAA-restricted diets in metabolic homeostasis

The consumption of BCAA in conjunction with a high-fat diet or a western diet induces insulin resistance in rodents, in part through the activation of mTORC1 activity^8,68^. Conversely, short-term deprivation of BCAAs improved glucose tolerance and insulin sensitivity in mice by reducing the hepatic mTORC1/AMPK ratio^69^. These studies suggest a potential role for BCAAs in the regulation of metabolic homeostasis at the mechanistic level. More physiological studies that utilized long-term partial deprivation of dietary BCAAs showed improved metabolic health in mice, including increased energy expenditure and decreased fat mass^70,71^.

As BCAA catabolism is impaired in individuals with obesity and insulin resistance^37,57,65^, it is plausible that a BCAA-restricted diet could improve disease conditions. Indeed, a BCAA-restricted diet reduced the accumulation of fatty acyl CoAs and increased muscle insulin sensitivity and energy expenditure in Zucker fatty rats^72^. Furthermore, in a related study, a BCAA-restricted diet in Zucker fatty rats improved cardiac metabolism by shifting fuel metabolism from glucose to fatty acids^73^. Interestingly, the beneficial effect of BCAA restriction on the mammalian heart does not alter the enzyme activity of BCKDH in the heart, suggesting that reduced uptake of BCAAs from circulation is critical.

Recently, the effect of a BCAA-restricted diet on metabolic health have been tested in clinical settings. In the first pilot study, a BCAA-restricted diet, in which the overall protein intake was not altered, reduced circulating BCAA levels by 50% in 7-day human trials^74^. Interestingly, the BCAA-restricted diet-fed group exhibited reduced plasma insulin levels, which is associated with improved insulin sensitivity, without changes in body weight in nonobese young individuals. These findings suggested that a BCAA-restricted diet might be beneficial for combating obesity and T2D in humans. In another randomized clinical trial, patients with well-controlled T2D consumed a BCAA-restricted diet for 4 weeks^75^. After this BCAA-restricted diet, the T2D patients experienced improved metabolic health, as indicated by an increased oral glucose sensitivity index, increased plasma fibroblast growth factor (FGF) 21 levels, reduced meal-derived insulin secretion, and decreased mTORC1 activity with improved mitochondrial respiratory capacity in adipose tissues in comparison to those in the placebo control group. Therefore, longer-term BCAA restriction studies that evaluate the safety and efficacy of this dietary regimen as an alternative strategy for the treatment of obesity and T2D in humans would be advantageous.

### Effects of a BCAA-restricted diet on promoting healthspan

Recently, BCAA limitation was shown to extend life span in Drosophila via histone modifications in neurons^76^. In mammals, a low-BCAA diet (approximately one-third of the BCAA content of a normal diet), especially a low-isoleucine diet, more effectively extended the lifespan of female flies than that of male flies. Mechanistically, the authors found that histone H3.3, a variant that accumulates in regions of active transcription, replaced histone H3 in the brain. In addition, consuming a low-BCAA diet reduced lysine 9 and lysine 27 acetylation of H3, which are markers of active transcription. These findings reveal a complex mechanism for transcriptional regulation in a specific subset of neurons in response to hunger and/or a BCAA-restricted diet. Interestingly, the amount of histone modification changes decreased during prolonged BCAA limitation in flies, suggesting that these complex changes in neuronal histone modifications might be critical for extending the lifespan of these animals.

mTORC1 activity was shown to be a major activator of mammalian aging via the induction of senescence, inhibition of autophagy, and stem cell exhaustion^77^, and BCAA can affect metabolic homeostasis via the regulation of the mTORC1 pathway. Thus, it was postulated that dietary restriction of BCAA could also affect longevity and/or healthspan in mammals. Indeed, a BCAA-rich diet impaired metabolic homeostasis and reduced lifespan in mice, showing that BCAAs could be detrimental to healthspan in mammals^8,9^. Conversely, prolonged partial dietary restriction of BCAAs improved overall health in mice, with a noticeable increase in the lifespan of progeria syndrome model mice^10^. Interestingly, while a BCAA-restricted diet also improved the healthspan of wild-type male mice, female mice fed the same diet did not exhibit changes in lifespan. These findings suggest that the potential effect of BCAAs on metabolic health or lifespan acts in a sex-specific manner.

Recently, we showed that aging specifically perturbs BCAA catabolism in adipose tissues by reducing the expression of key regulatory proteins in this pathway^78^. BCAA catabolism was significantly lower in the white adipose tissue (WAT) of aged mice than in that of young control mice. Mechanistically, an age-associated increase in CREB-regulated transcription coactivator (CRTC) 2 led to enhanced expression of HES-1. This increased expression reduced PPARγ expression and subsequently decreased the expression of BCAA catabolic enzymes in WAT. Increased BCAA levels activate the mTORC1 pathway. This leads to enhanced cellular senescence, increased proinflammatory macrophage levels, and decreased adipogenic progenitor cell numbers within WAT, which results in the promotion of metabolic disorders in mice. Interestingly, genetic depletion of CRTC2 in adipocytes reversed age-associated metabolic disorders in mice in part by restoring the expression of BCAA catabolic enzymes in WAT. These findings suggest that intervention in the age-associated perturbation of BCAA catabolism in adipose tissue could be beneficial for maintaining the healthspan of mammals. Confirmation of this finding in humans is necessary before pursuing the development of specific agents that increase the expression of BCAA catabolic enzymes in adipose tissue as potential therapeutic agents for combating age-associated metabolic disorders.

### Effects of individual BCAAs on controlling healthspan

While the initial stages of BCAA breakdown involve common enzymes, such as BCAT and BCKDH, the subsequent catabolic intermediates for each of the three BCAAs are different from one another. Thus, it is plausible that each BCAA could function differently. For example, 3-HIB, an intermediate of valine catabolism, is specifically associated with lipotoxicity in skeletal muscle^66^. Thus, the effects of individual BCAAs on healthspan could be distinct from each other.

To test the effects of a specific BCAA-restricted diet on metabolic health, individual restriction of each BCAA was tested in mice^70,71^. Surprisingly, 3 months of dietary restriction of isoleucine or valine, but not leucine, improved metabolic health in mice, as shown by increased glucose tolerance and insulin sensitivity in mice. Furthermore, a low-isoleucine diet promoted increased beige adipogenesis and energy expenditure, in part by enhancing FGF21 expression in the liver and adipose tissues. Finally, metabolic disorders induced by a Western diet in mice were attenuated by deprivation of isoleucine and, to a somewhat lesser extent, valine. These findings suggest that the beneficial effect of a BCAA-restricted diet on metabolic health might be mediated mainly by deprivation of isoleucine in the diet.

Finally, the effects of an isoleucine-restricted diet on longevity and healthspan were compared with those of a BCAA-restricted diet in a recent study^79^. To better reflect the genetically diverse human population, the authors used the genetically heterogeneous UM-HET3 mice. Compared with the control diet, lifelong feeding of an isoleucine-restricted diet improved the healthspan of the mice, as shown by reduced fat mass, improved glycemic control, and increased energy expenditure. Furthermore, isoleucine-restricted diet feeding enhanced the longevity of male mice and, to a lesser extent, of female mice. As stated in the previous section, a BCAA-restricted diet only extended the lifespan of male mice but not of female mice. Thus, an isoleucine-restricted diet might be a better dietary strategy than a BCAA-restricted diet for potentially enhancing the longevity and healthspan of humans.

## Concluding remarks

The role of BCAAs and their metabolism in metabolic health and lifespan has been extensively studied recently. These studies were mainly conducted in model organisms, including rodents, due in part to the convenience of obtaining mechanistic insights by using genetically modified animal models. Furthermore, most studies were conducted on male animals. As shown in a recent report, there were sex differences in the effects of a BCAA-restricted diet on extending the lifespan of mice, suggesting that the effect of BCAA catabolism or its downstream signaling pathway should be more carefully analyzed in both sexes in future experiments. The potential beneficial effect of BCAA supplementation should also be more carefully analyzed in both sexes, with in-depth evaluation of metabolic pathways, downstream signaling pathways, and physiological and pathological consequences in distinct tissues. Future studies will be necessary to elucidate the mechanisms underlying the apparent beneficial effect of BCAA restriction, specifically isoleucine restriction, on metabolic health and lifespan, which could eventually lead to the development of drugs that extend the healthspan of humans.
